# Dietary Addition of Antioxidant Complex Packs and Functional Amino Acids Can Improve the Digestion, Absorption, and Immunity of Huanjiang Minipigs

**DOI:** 10.1155/2020/1475831

**Published:** 2020-09-29

**Authors:** Zhijuan Cui, Bie Tan, Simeng Liao, Ming Qi, Xianze Wang, Andong Zha, Xiangfeng Kong, Yuguang Chen, Peng Liao

**Affiliations:** ^1^Hunan Agricultural University, Changsha 410128, China; ^2^Laboratory of Animal Nutritional Physiology and Metabolic Process, Key Laboratory of Agro-Ecological Processes in Subtropical Region, National Engineering Laboratory for Pollution Control and Waste Utilization in Livestock and Poultry Production, Institute of Subtropical Agriculture, Chinese Academy of Sciences, Changsha, 410125 Hunan, China

## Abstract

To study the effect of functional amino acids and the antioxidant function compound package on Huanjiang minipigs and to lay a foundation for the formulation of green and efficient feed for Huanjiang minipigs, we added functional amino acids and the antioxidant function compound package to piglet feed for 28 days. After feeding, we detected the growth performance, biochemical indexes, inflammatory indexes, and intestinal disaccharidase of piglets. It was found that functional amino acids and the antioxidant compound package had certain effects on the growth performance and biochemical indexes of piglets and could reduce the level of IL-6 and increase the level of LZM and SIgA of piglets, and the levels of lactase and maltase in the intestine also increased significantly. The results showed that the compound package of functional amino acids and antioxidation could improve the growth performance and immunity of piglets and promote the digestion and absorption of nutrients in piglets.

## 1. Introduction

The Huanjiang minipig is a famous small local pig breed in China, which has the characteristics of strong stress resistance, rough feeding resistance, good meat quality, and so on [1]. In addition, because Huanjiang minipigs have been feeding roughage and green feed for a long time, there is no unified feeding standard and scientific feed formula. However, weaning piglets are faced with a variety of external environmental factors such as feed and environmental stress, which often lead to disorder of digestive function, damage of the intestinal barrier, diarrhea, and slow growth [2, 3]. Thus, it not only affects the interests of farmers but also reduces the output of commercial pigs. Therefore, it is important to regulate the nutrition of piglets during weaning.

Over the years, the problem of drug resistance and drug residues caused by antibiotics is becoming more and more serious, which has aroused widespread concern among consumers. The European Union has banned the use of antibiotics as a feed additive [4]. Our antioxidant package contains a variety of active ingredients, including functional amino acids (guanidinoacetic acid, L-glutamic acid, etc.), functional trace elements (yeast selenium, zinc methionine chelate, etc.), traditional chinese medicine extracts (baicalin, grape seed proanthocyanidins, etc.), vitamins (vitamin A, vitamin C, etc.), and functional polysaccharides (yeast polysaccharides, astragalus polysaccharides, etc.). These are some common nutritional additives, which can effectively promote the growth of piglets. Yeast selenium can effectively alleviate the growth inhibition of piglets caused by lipopolysaccharide [5], baicalin can significantly reduce the diarrhea rate of piglets [6], and astragalus polysaccharides can promote the digestion and absorption of piglets. Vitamin A plays an important role in maintaining the integrity of the intestinal mucosal epithelial barrier, regulating mucosal immune response and anti-infection. Deficiency of vitamin A and vitamin C can cause nutritional and metabolic diseases in weaned piglets. Glutamine, glutamate, and aspartate have been proven to have good effects on weaned piglets in production practice [7–9]. Recent studies have shown that glutamine is rich in amino acids in physiological fluids and proteins and is the key regulator of gene expression [10]. In the previous study, the addition of glutamine significantly reduced the diarrhea rate of piglets during weaning, indicating that glutamine can effectively regulate the physiological state of piglets' intestines [11]. Xiao et al. used gas chromatography to study the effects of glutamine on nutrient metabolism in piglets. They found that carbohydrates, amino acids, fatty acids, and other substances were significantly different [12]. Glutamate and aspartic acid are functional amino acids, which play a variety of functions in nutrient metabolism, immune response, signal pathway regulation, and synaptic transmission [7–9]. Previous studies have shown that glutamine, glutamate, and aspartate can improve the growth performance of piglets and reduce the ratio of feed to meat; also, glutamate can improve the oxidative stress induced by diquat and improve the expression of amino acid transporter [13, 14]. And oral administration of 0.5 g/kg BW glutamate to suckling piglets can effectively improve intestinal morphology and affect the metabolism of amino acids in piglets and increase the content of VFA in the cecum and colon [15]. Pia et al. found that the intestinal injury of piglets caused by lipopolysaccharide could be alleviated by adding 0.5% aspartic acid, and the intestinal energy state could be effectively improved by increasing ADP and ATP and reducing the ratio of AMP/ATP [16]. We tried to combine these nutrients together to study the effect on the growth performance of Huanjiang minipigs.

Therefore, to study the effects of the dietary antioxidant complex package and complex ligands of glutamate, glutamine, and aspartic acid on growth performance, biochemical indexes, cytokines, and intestinal enzyme activity of Huanjiang minipigs, we aim to provide a theoretical basis for the development of efficient feed additives for Huanjiang minipigs.

## 2. Materials and Methods

### 2.1. Glutamine, Glutamate, Aspartate, and Antioxidant Compound Package

Glutamine, glutamate, aspartate, and the antioxidant compound package components are purchased from Fujian Longyan Tai Mai Sanluo Pharmaceutical Co., Ltd. At the beginning of the formal trial, the nutrients were mixed in a percentage into the basic diet. No antibiotics were added to all diets. The basic dietary composition and nutrient level are shown in Table 1. The composition of the antioxidant compound package is shown in Table 2.

### 2.2. Animals and Experimental Design

This study was conducted in accordance with the guidelines of the Institute of Subtropical Agriculture, Chinese Academy of Sciences. All experimental protocols were approved by the animal ethical committee of the Institute of Subtropical Agriculture, Chinese Academy of Sciences (2013020).

A total of 96 healthy Huanjiang minipigs (age 21 d) were used in this study. They were randomly assigned to 4 treatments, with six pens per treatment and four piglets per pen. The four treatment groups were as follows: basic diet group (CON), basic diet+3.5 mg/kg antioxidant compound package (ACP), basic diet+1% glutamine+0.5% glutamate+0.1% aspartate (FAA), and basic diet+3.5 mg/kg antioxidant compound package+1% glutamine+0.5% glutamate+0.1% aspartate (ACP+FAA). Piglets, 3 days after adoption, were fed four times per day at 7:30 am, 11:30 am, 2:30 pm, and 6:00 pm for a 28-day period after. The experiment was carried out indoors, and the activity space met the action of piglets, and piglets were free to eat and drink water. The experimental basic diet was designed to meet the national research council pig nutrition requirements (NRC, 1998) and Chinese pig feeding standards (NY/T 65-2004) [17].

In the morning of day 28, the final body weight, average daily feed intake (ADFI), and average daily gain (ADG) were collected. After the experiment, one piglet was randomly selected from each pigpen and killed, and then, the serum was collected in the morning. After electrically slaughtering, jejunum (2 cm) and ileum (2 cm) samples were immediately and rapidly excised with ice-cold physiological saline. Some samples were then either placed in liquid or stored at −80°C for further analysis. And some samples of the jejunum and ileum were stored in formaldehyde solution until further analysis.

### 2.3. Serum Biochemical Index Assays

Biochemical indicators (triglyceride, blood ammonia, D-lactate, and low-density lipoprotein cholesterol) were measured using an instrument (Biochemical Analytical Instrument, Beckman CX4, Beckman Coulter Inc., Brea, CA) and commercial kits (Sino-German Beijing Leadman Biotech Ltd., Beijing, China).

### 2.4. Determination of Serum Cytokines

Plasma concentrations of interleukin-1*β* (IL-1*β*), IL-4, IL-6, IL-8, IL-10, tumor necrosis factor alpha (TNF-*α*), transformed growth factor beta (TGF-*β*), and interferon gamma (IFN-*γ*) were determined using the gene chip method. A capture antibody is first bound to the glass surface. After incubation with the sample, the target cytokine is trapped on the solid surface. A secondary biotin-labeled detection antibody is then added, which can recognize a different epitope of the target cytokine. The cytokine-antibody-biotin complex can then be visualized through the addition of the streptavidin-conjugated Cy3 equivalent dye, using a laser scanner.

### 2.5. Determination of Intestinal Enzyme Activity

Jejunum and ileum concentrations of lactase, invertase, maltase, lysozyme (LZM), beta defensins (*β*-PBD), and secretory IgA (SIgA) were measured using commercially available swine enzyme-linked immunosorbent assay (ELISA) kits according to the manufacturer's instructions (Meimian Industrial Co., Ltd., Jiangsu, China).

### 2.6. Statistical Analyses

Data were analyzed by the analysis of variance, using the general linear model procedure of SPSS 20.0 (SPSS Inc., Chicago, IL, USA). The differences among treatments were evaluated using one-way ANOVA with Tukey's test. Results were expressed as the mean ± standard error of mean (SEM). A value of *P* < 0.05 was considered statistically significant, whereas 0.05 < *P* < 0.1 was considered a trend.

## 3. Results

### 3.1. Growth Performance

Table 3 summarizes the performance of piglets given antioxidant composite packs and functional amino acids and antioxidant composite packs and functional amino acid mixtures. Compared with CON, ADG of piglets in the ACP+FAA group was significantly increased and F/G of piglets was significantly decreased (*P* < 0.05). There was no significant difference in ADG, ADFI, and F/G between CON, ACP, and FAA groups (*P* > 0.05).

### 3.2. Serum Biochemical Indexes

As shown in Table 4, the analysis of serum biochemical results showed that the D-lactate (D-LACT) levels of the ACP, FAA, and ACP+FAA groups were significantly lower than those of the CON group (*P* < 0.05). In addition, the LDL-C3 levels of the ACP group were significantly decreased compared with those of the CON group (*P* < 0.05). Moreover, serum concentrations of LDL-C3 in the ACP+FAA group had a tendency to decrease compared to those in the CON group (0.1 < *P* < 0.05).

### 3.3. Plasma Cytokines

**Figure 1:**
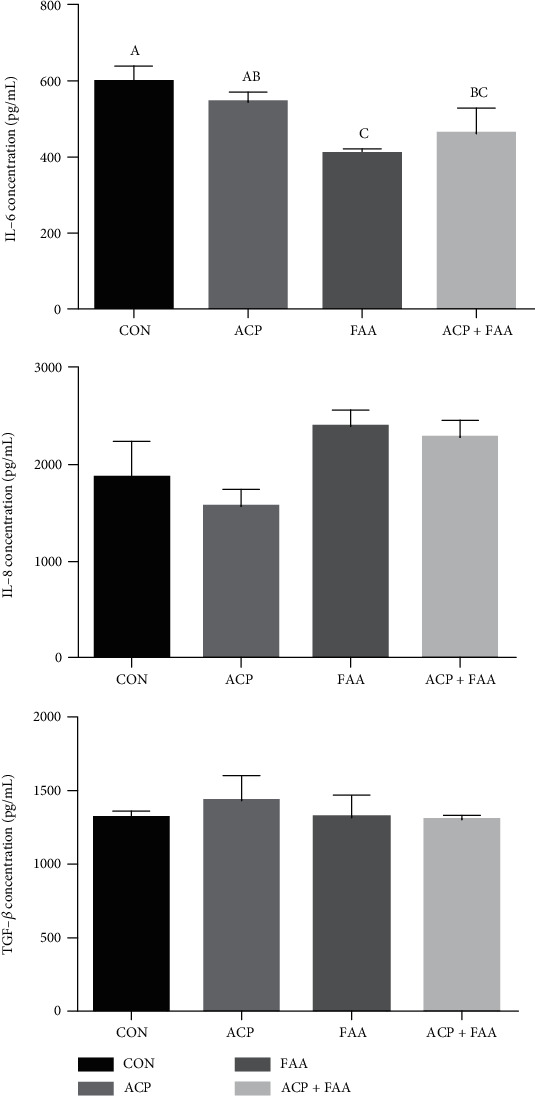
Cytokine concentration of IL-6, IL-8, and TGF-*β* in serum of Huanjiang minipigs. Data are expressed as means ± SEM. *n* = 6. ^∗^*P* < 0.05 versus healthy pigs. Means without a common letter differ (*P* < 0.05). CON: basic diet group; ACP: basic diet+3.5 mg/kg antioxidant compound package; FAA: basic diet+1% glutamine+0.5% glutamate+0.1% aspartate; ACP+FAA: basic diet+3.5 mg/kg antioxidant compound package+1% glutamine+0.5% glutamate+0.1% aspartate.

**Figure 2:**
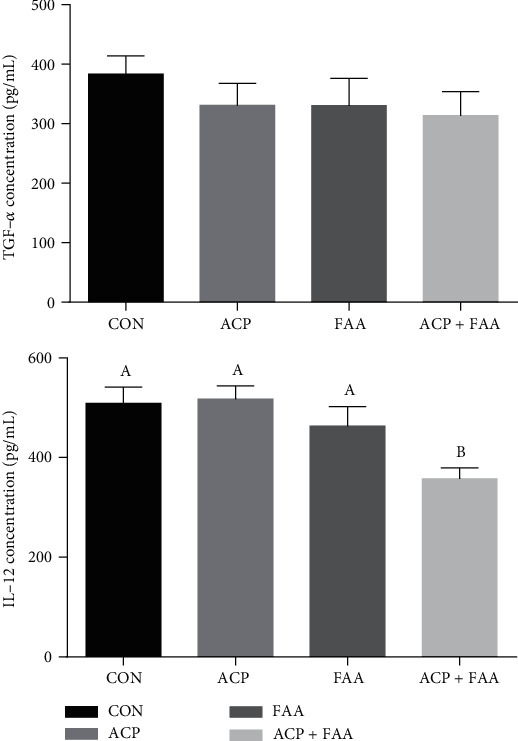
Cytokine concentration of TNF-*α* and IL-12 in serum of Huanjiang minipigs. Data are expressed as means ± SEM. *n* = 6. ^∗^*P* < 0.05 versus healthy pigs. Means without a common letter differ (*P* < 0.05). CON: basic diet group; ACP: basic diet+3.5 mg/kg antioxidant compound package; FAA: basic diet+1% glutamine+0.5% glutamate+0.1% aspartate; ACP+FAA: basic diet+3.5 mg/kg antioxidant compound package+1% glutamine+0.5% glutamate+0.1% aspartate.

As shown in Figures 1 and 2, serum concentrations of IL-12 in the ACP+FAA group were significantly lower than those in the CON group (*P* < 0.05). Significantly lower concentration of IL-6 in serum was found in the FAA and ACP+FAA groups (*P* < 0.05). And serum concentrations of IL-8, TNF-*α*, and TGF-*β* have no difference between the four groups.

### 3.4. Determination of Intestinal Enzyme Activity

**Figure 3:**
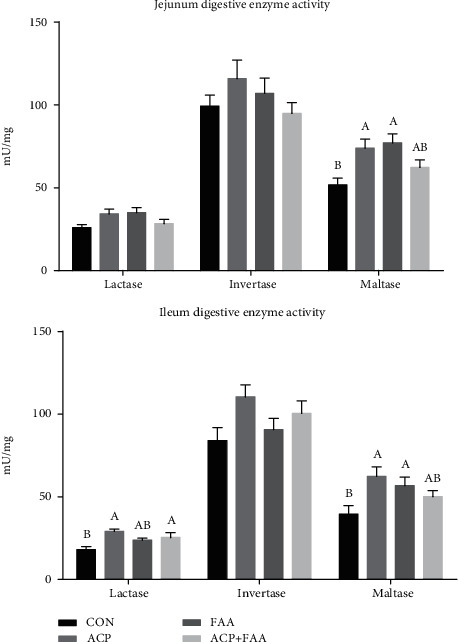
Concentration of lactase, invertase, and maltase in the jejunum and ileum of Huanjiang minipigs. Data are expressed as means ± SEM. *n* = 6. ^∗^*P* < 0.05 versus healthy pigs. Means without a common letter differ (*P* < 0.05). CON: basic diet group; ACP: basic diet+3.5 mg/kg antioxidant compound package; FAA: basic diet+1% glutamine+0.5% glutamate+0.1% aspartate; ACP+FAA: basic diet+3.5 mg/kg antioxidant compound package+1% glutamine+0.5% glutamate+0.1% aspartate.

The concentrations of lactase, invertase, and maltase in the jejunum and ileum are shown in Figure 3. In the jejunum, the maltase of groups ACP and FAA was significantly higher than that of group CON (*P* < 0.05). In the ileum, the ACP and ACP+FAA diet pigs showed higher serum concentrations of lactase compared with the CON group (*P* < 0.05), and the ACP and FAA groups showed increased concentration of maltase (*P* < 0.05).

**Figure 4:**
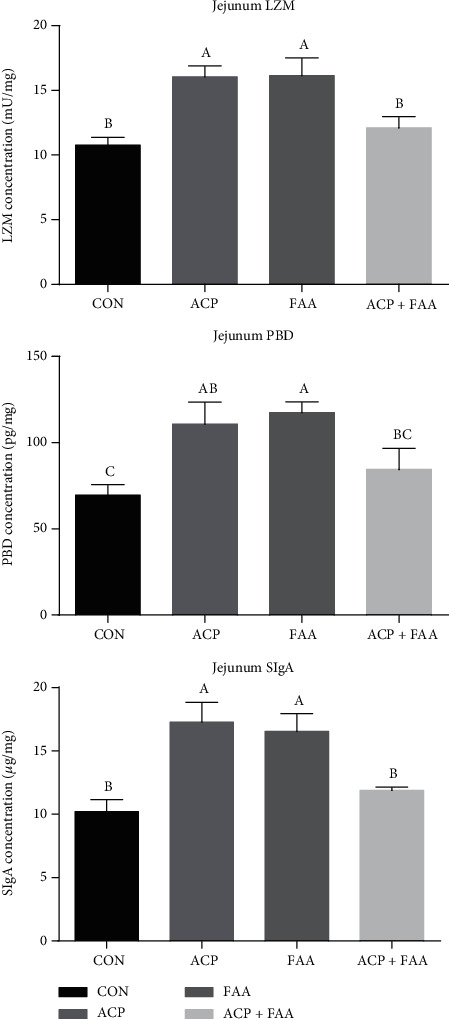
Concentration of LZM, *β*-PBD, and SIgA in the jejunum of Huanjiang minipigs. Data are expressed as means ± SEM. *n* = 6. ^∗^*P* < 0.05 versus healthy pigs. Means without a common letter differ (*P* < 0.05). CON: basic diet group; ACP: basic diet+3.5 mg/kg antioxidant compound package; FAA: basic diet+1% glutamine+0.5% glutamate+0.1% aspartate; ACP+FAA: basic diet+3.5 mg/kg antioxidant compound package+1% glutamine+0.5% glutamate+0.1% aspartate.

**Figure 5:**
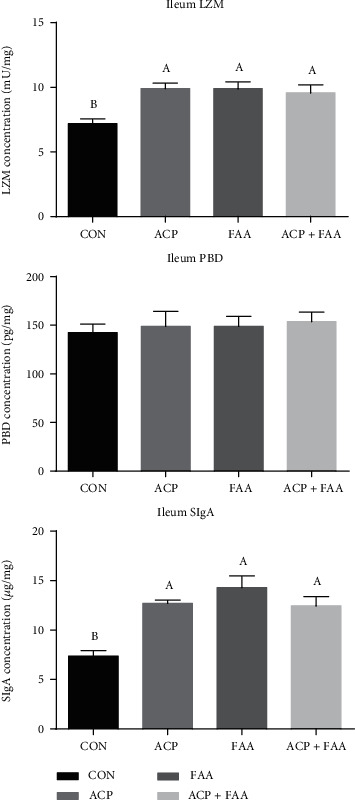
Concentration of LZM, *β*-PBD, and SIgA in the ileum of Huanjiang minipigs. Data are expressed as means ± SEM. *n* = 6. ^∗^*P* < 0.05 versus healthy pigs. Means without a common letter differ (*P* < 0.05). CON: basic diet group; ACP: basic diet+3.5 mg/kg antioxidant compound package; FAA: basic diet+1% glutamine+0.5% glutamate+0.1% aspartate; ACP+FAA: basic diet+3.5 mg/kg antioxidant compound package+1% glutamine+0.5% glutamate+0.1% aspartate.

The concentrations of LZM, *β*-PBD, and SIgA in the jejunum and ileum are shown in Figures 4 and 5. Compared with the CON group, the ACP and FAA groups showed significantly increased concentrations of LZM, *β*-PBD, and SIgA in the jejunum (*P* < 0.05). There was no significant difference between the ACP+FAA group and the CON group, but *β*-PBD in the ACP+FAA group tended to increase compared with that in the CON group (*P* > 0.05). In the ileum, the SIgA and LZM of groups ACF, FAA, and ACF+FAA were significantly increased compared with those of the CON group.

## 4. Discussion

### 4.1. The Effect of ACP, FAA, and ACP+FAA on Growth Performance of Huanjiang Minipigs

After weaning, due to the effects of environmental changes and weaning stress, it is easy to cause intestinal mucosal damage and morphological changes in piglets, thus reducing the growth performance of piglets. Therefore, it is very important to add nutrients during weaning. Glutamine can be effectively used in the small intestine and can repair intestinal mucosal damage caused by weaning stress [18]. Glutamine is the main source of intestinal cells; in fact, glutamate and aspartic acid can also be used as fuel to provide energy to the intestine and so does glutamine [7]. Stoll et al. injected glutamate into the intestine of piglets. The results showed that most of the glutamate could be digested and utilized by the intestine [19]. Therefore, glutamine, glutamic acid, and aspartic acid can effectively improve the growth performance of piglets [13, 14]. In this experiment, we used glutamine, glutamic acid, and aspartic acid in proportion, although they did not significantly improve the growth performance of piglets, but the average daily gain increased and the ratio of feed to meat decreased. The main components of the antioxidant compound package have antibacterial and antioxidation effects, improving immunity and the intestinal barrier [20–23]. However, there was no significant difference between the ACP group and the control group, which may be due to the fact that the proportion of various components in the antioxidant complex package did not reach the best performance, so it could not improve the growth performance of piglets. It is worth noting that the mixed use of two kinds of treatments in the ACP+FAA group can significantly improve the growth performance of Huanjiang minipigs, so we speculate that there is a synergistic effect between amino acids and the antioxidant compound package, which needs to be further studied.

### 4.2. The Effect of ACP, FAA, and ACP+FAA on Serum Biochemistry of Huanjiang Minipigs

D-LACT is a special bacterial metabolite produced by the intestinal tract [24]. When intestinal injury leads to the exfoliation of the apical epithelium of the intestinal mucosa and the injury of intestinal epithelial tight junction leads to the increase in intestinal mucosal permeability, a large amount of D-LACT produced by bacteria in the intestinal tract enters the blood through the damaged mucosa, which will increase the level of plasma D-LACT. The current data show that the ACP, FAA, and ACP+FAA treatments can all reduce the level of serum D-LACT, suggesting that these three treatments can improve the intestinal health of Huanjiang minipigs to some extent. Low-density lipoprotein cholesterol is one of the low-density lipoproteins, which can cause atherosclerosis when the content is too high [25]. And a large number of studies have shown that lowering plasma total cholesterol and low-density lipoprotein cholesterol (LDLC) can reduce the risk of cardiovascular death, myocardial infarction, and stroke [26]. In our experiment, the level of LDL-C3 was significantly decreased in the ACP group, and LDL-C3 has a tendency to decrease in the ACP+FAA group.

### 4.3. The Effect of ACP, FAA, and ACP+FAA on Serum Porcine Cytokines in Huanjiang Xiang Pigs

Cytokines are a kind of small molecular proteins with a wide range of biological activities synthesized and secreted by immune cells (such as monocytes, macrophages, T cells, B cells, and NK cells) and some nonimmune cells (endothelial cells, epidermal cells, etc.). Cytokines include interleukin, interferon, tumor necrosis factor superfamily, and other categories, and these cytokines play an important role in regulating immunity. Among them, IL-1, IL-6, IL-8, and TNF-*α* are important proinflammatory factors [27]. The expression of IL-8 is regulated by transcription factor nuclear factor-*κ*b (NF-*κ*B). According to the previous study, we found that glutamine could decrease the activity of NF-*κ*B, and the level of IL-8 in the jejunum was also significantly decreased [28, 29]. However, although IL-8 has an upward trend in this study, the result is not significant. At present, it is believed that the inflammatory response induced by IL-6 and TNF-*α* is largely achieved by inducing the production of chemokines represented by IL-8 [30]. It has been found that IL-6 can regulate inflammatory hypoxemia by inducing the synthesis of the iron-regulating hormone hepcidin [31]. In this study, the addition of FAA or ACP+FAA significantly reduced the content of IL-6 in the serum of piglets, and the group of ACP also had a downward trend, which significantly reduced the inflammatory response of piglets. IL-12 is also an important inflammatory factor, which can promote interferon production and induce CD4^~+^T cells to differentiate into Th1 (T helper cell) cells. In this experiment, the mixed additives of functional amino acids and the antioxidant compound package significantly reduced the level of IL-12 in serum.

### 4.4. The Effect of ACP, FAA, and ACP+FAA on Intestinal Enzyme Activity in Huanjiang Xiang Pigs

The intestinal tract of piglets contains many different enzymes; among which, disaccharide hydrolase (sucrase, maltase, and lactase) can convert different polysaccharides into glucose and other monosaccharides, providing energy for piglets. Therefore, it plays an important role in the growth and development of piglets. Generally speaking, the activity of these enzymes in the intestine increases gradually as the piglets grow but decreases inversely during weaning. In the Douglas study, the addition of glutamine and glutamate did not increase the activity of disaccharidase in piglets [32]. But 1% glutamine could increase the activities of sucrase, maltase, and lactase in the jejunal mucosa of piglets [33]. Consistently, the sucrase in the duodenum and maltase in the duodenum and jejunum of piglets fed with mixed diet of arginine and glutamine were also higher than those of the control group [34]. This is consistent with our results that lactase and maltose in the jejunum and ileum were significantly increased in the group with the antioxidant complex and functional amino acids, which indicated that the antioxidant complex and functional amino acids could promote the digestive function of the small intestine and promote the growth of piglets.

### 4.5. The Effect of ACP, FAA, and ACP+FAA on SIgA, PBD, and LZM in Huanjiang Xiang Pigs

Secretory IgA (SIgA) plays an important role in protecting body health. As the first line of protection for the intestinal epithelium, it protects the intestinal epithelial cells from enterotoxins and pathogenic microorganisms [35]. SIgA prevents microorganisms and toxins (cholera toxin, Shiga toxin, etc.) from attaching to surface epithelial cells and blocks contact between them [36]. Defensin is a group of antimicrobial peptides found in different organisms, and *β*-defensin is one of them. *β*-Defensins have antimicrobial activity against Gram-negative bacteria and fungi by interacting with the outer surface of microorganisms [37]. In addition, *β*-defensin has chemotactic activity on T cells and dendritic cells and can induce monocytes and epithelial cells to produce cytokines [38]. Lysozyme is an alkaline enzyme that can hydrolyze mucopolysaccharides in pathogenic bacteria and can destroy the *β*-1~4 glycosidic bond between N-acetylteichoic acid and N-acetylglucosamine in the cell wall [39]. Adding LZM to diet can effectively promote the development of the intestinal structure and function and promote the enrichment of beneficial microorganisms in intestinal flora [40]. In this study, the three groups supplemented with ACP, FAA, and ACP+FAA had significantly increased levels of LZM and SIgA in the ileum, and the two groups supplemented with ACP and FAA also had significantly increased levels of SIgA, PBD, and LZM in the jejunum, suggesting that ACP, FAA, and ACP+FAA played a positive role in intestinal immunity of Huanjiang minipigs.

## 5. Conclusion

Diets supplemented with ACP+FAA can improve the growth performance of Huanjiang minipigs to a certain extent. And ACP, FAA, and ACP+FAA could reduce the IL-6 in serum and increase the activity of disaccharidase and the level of LZM and *β*-PBD in the intestinal tract of piglets, which promoted the absorption of nutrients and increased the resistance of piglets.

## Figures and Tables

**Table 1:** Basic dietary composition and nutrient level.

Ingredient	Content	Nutrient level	Content
Corn	55.5	DE	13.5
Soybean meal	22	CP	17
Wheat bran	10.13	Lys	1.08
Rice bran	8.37	Met+Cys	0.62
4% premix	4	Thr	0.69
Total	100	Ca	0.74
		TP	0.58

Premix was provided per kilogram of diet. Per kilogram of diet: VA 12000 IU, VD_3_ 2500 IU, VE 30 IU, VK_3_ 3 mg, VB_12_ 18 *μ*g, riboflavin 4 mg, nicotinic acid 40 mg, pantothenic acid 15 mg, choline chloride 400 mg, folic acid 700 *μ*g, VB_1_ 1.5 mg, VB_6_ 3 mg, biotin 100 *μ*g, Zn 80 mg, Mn 20 mg, Fe 83 mg, Cu 25 mg, IQ 48 mg, and Se Q 36 mg.

**Table 2:** Composition of the antioxidant composite package.

	Component	Proportion
Functional amino acids	Guanidinoacetic acid	0.7
	L-Glutamic acid	0.5
	L-Methionine	0.5

Functional trace element	Selenium yeast	3
	Zn-Met2 chelate	1.5
	Seleno-DL-methionine	1

Chinese herb extracts	Baicalin	2
	Grape seed proanthocyanidin	1.2
	Artichoke extract	1.3

Vitamins	Vitamin A	1
	Vitamin C	1
	Vitamin E	3

Functional polysaccharide	Zymosan	4
	Astragalus polysaccharides	5
	Echinacea polysaccharide	2.3

Probiotics	*Lactobacillus*	0.5
	*Bacillus subtilis*	4
	*Enterococcus faecium*	0.5

Isotopic carrier	Butabalus (raw powder)	3
	White carbon black	64
	Total	100

**Table 3:** Growth performance of Huanjiang minipigs.

	CON	ACP	FAA	ACP+FAA	*P*
Initial weight (kg)	4.26 ± 0.23	4.41 ± 0.28	4.57 ± 0.58	4.61 ± 0.68	0.054
Final weight (kg)	7.61 ± 0.52^b^	7.33 ± 0.38^b^	7.61 ± 0.59^b^	8.37 ± 0.28^a^	0.006
Average daily gain (g/day)	114.64 ± 22.44^b^	110.21 ± 8.56^b^	118.81 ± 8.42^ab^	132.92 ± 8.19^a^	0.044
Average daily feed intake (g/day)	247.58 ± 17.07	247.58 ± 14.05	250.64 ± 21.56	273.27 ± 22.06	0.086
Feed/gain (g/g)	2.59 ± 0.31^a^	2.28 ± 0.22^ab^	2.31 ± 0.30^ab^	2.30 ± 0.21^b^	0.016

^a,b^Values within a row with different superscripts differ significantly (*P* < 0.05). CON: basic diet group; ACP: basic diet+3.5 mg/kg antioxidant compound package; FAA: basic diet+1% glutamine+0.5% glutamate+0.1% aspartate; ACP+FAA: basic diet+3.5 mg/kg antioxidant compound package+1% glutamine+0.5% glutamate+0.1% aspartate.

**Table 4:** Blood biochemical indexes of Huanjiang minipigs.

	CON	ACP	FAA	ACP+FAA	*P*
D-LACT (mmol/L)	21.58 ± 0.45^a^	21.10 ± 0.00^b^	21.11 ± 0.00^b^	21.11 ± 0.00^b^	<0.05
TG (mmol/L)	1.27 ± 0.18	1.29 ± 1.59	1.22 ± 0.42	0.80 ± 0.22	0.14
NH_3_ (*μ*mol/L)	387.46 ± 95.15	270.53 ± 86.32	248.56 ± 59.32	292.66 ± 119.92	0.08
LDL-C3 (mmol/L)	1.49 ± 0.42^a^	1.09 ± 0.14^b^	1.46 ± 0.19^a^	1.23 ± 0.16^ab^	0.042

^a,b^Values within a row with different superscripts differ significantly (*P* < 0.05). CON: basic diet group; ACP: basic diet+3.5 mg/kg antioxidant compound package; FAA: basic diet+1% glutamine+0.5% glutamate+0.1% aspartate; ACP+FAA: basic diet+3.5 mg/kg antioxidant compound package+1% glutamine+0.5% glutamate+0.1% aspartate.

## Data Availability

All data generated or analyzed during this study are included in this published article.

## Abbreviations

TG: Triglyceride
NH3: Blood ammonia
D-LACT: D-Lactate
LDL-D3: Low-density lipoprotein cholesterol
IL-1*β*: Interleukin-1*β*
IL-4: Interleukin-4
IL-6: Interleukin-6
IL-8: Interleukin-8
IL-10: Interleukin-10
LZM: Lysozyme
SIgA: Secretory IgA
*β*-PBD: *β*-Defensins
TNF-*α*: Tumor necrosis factor alpha
TGF-*β*: Transformed growth factor beta
IFN-*γ*: Interferon gamma.
